# Potent cellular immune responses after therapeutic immunization of HIV-positive patients with the PENNVAX®-B DNA vaccine in a Phase I Trial

**DOI:** 10.1186/1742-4690-9-S2-P276

**Published:** 2012-09-13

**Authors:** P Tebas, L Ramirez, MP Morrow, J Yan, D Shah, J Lee, DB Weiner, J Boyer, M Bagarazzi, NY Sardesai

**Affiliations:** 1 Department of Pathology and Laboratory Medicine, U of Pennsylvania, Philadelphia, PA, USA; 2 Inovio Pharmaceuticals, Blue Bell, PA, USA

## Background

Although highly active antiretroviral therapy (HAART) regimens have dramatically transformed treatment of HIV infection, achieving 95% adherence to HAART regimens is notoriously difficult. The successful creation of an immunotherapy for infection could eliminate the potential pitfalls associated with the necessity for long-term adherence to drug therapy. To that end, we evaluated the safety and immunogenicity of the PENNVAX®-B vaccine, delivered with in vivo electroporation (EP) in HIV-infected volunteers on HAART in a Phase I open-label study.

## Methods

Enrollment criteria included HIV RNA<75copies/mL, CD4>400/µL with nadir >200/µL. Twelve eligible subjects received a 4 dose series (day 0, weeks 4, 8 and 16) of 3 mg PENNVAX®-B (consisting of SynCon® HIV Gag, Pol, and Env immunogens) intramuscularly followed by in vivo EP with the CELLECTRA®-5P device.

## Results

All the enrolled subjects completed the immunization schedule. The vaccine demonstrated an acceptable safety profile and was generally well tolerated. Overall, 9 out of 12 subjects (75%) showed significant vaccine-specific T-cell responses in the form of IFN-γ ELISpot against at least one of the three vaccine antigens (Gag, Pol, or Env) following vaccination. Furthermore, responses were not dominated by a single antigen, as 50% of subjects had strong vaccine induced responses to at least 2 of the 3 antigens and 3 showed vaccine-induced responses to all 3 antigens. Importantly, the ELISpot responses induced by vaccination were predominantly CD8+T-cells, which are considered to be paramount in clearing chronic viral infections and an important measure of the performance of a therapeutic vaccine. Additionally, HIV-specific immune responses were assayed by flow cytometry to measure IFN-γ production by both CD4+ and CD8+T cells as well as co-expression of the CTL-related markers CD107a, GranzymeB and Perforin.

## Conclusion

Analysis of these data should provide more definitive evidence of HIV-specific CTL function, which has been implicated in control of viral replication in HIV-infected patients.

